# Effects of intracerebroventricular injection of corticotrophin releasing factor on the gene expression of ghrelin and corticotrophin releasing factor receptors in broiler chickens

**DOI:** 10.5713/ab.22.0046

**Published:** 2022-06-24

**Authors:** Yuanli Cai, Zhigang Song

**Affiliations:** 1 College of Life Science, Qilu Normal University, Jinan, Shandong 250200, China; 2 Department of Animal Science, Shandong Agricultural University, Taian, Shandong 271018, China

**Keywords:** Broiler Chickens, Ghrelin, Corticotropin-releasing Factor (CRF), Feed Intake

## Abstract

**Objective:**

This study aimed to investigate the effects of corticotropin-releasing factor (CRF) on the feed intake of broiler chickens and explore its influencing mechanism.

**Methods:**

The study included two trials. In trial 1, 32 male broiler chickens (Arbor Acres, *Gallus gallus domesticus*) were given ventricle buried tubes, and they were allowed to recover for 3 days. At 8:00 AM, intracerebroventricular (ICV) injection with CRF or normal saline was performed in 10-day-old broiler chickens, which were divided into the 5, 10, and 20 μg and control (normal saline) groups according to the dose of CRF injection. In trial 2, chickens were divided into the 10 μg and control group (physiological saline) to repeat trial 1.

**Results:**

Results of trial 1 showed that the cumulative amount of feed intake in the 10 or 20 μg groups was considerably lower than that of the control group after ICV injection with CRF. The lowest amount of feed intake was obtained with the addition of 10 μg of CRF. In trial 2, the expression of ghrelin in the hypothalamus injected with 10 μg of CRF increased significantly, but the expression of ghrelin in various sections of the small intestine considerably decreased. The expression of CRF receptor subtypes 1 (CRFR1) in the hypothalamus and some parts of the small intestine remarkably increased, and the expression of CRF receptor subtypes 2 (CRFR2) increased only in the duodenum, whereas the expression of growth hormone secretagogue receptor (GHSR-1α) in the jejunum and ileum increased considerably after ICV injection of 10 μg of CRF.

**Conclusion:**

The CRF at 10 μg increased ghrelin expression in the hypothalamus and CRFR1 expression in the small intestine, and this phenomenon was related to the suppressed feed intake of broiler chickens.

## INTRODUCTION

Corticotropin-releasing factor (CRF), a peptide consisting of 41 amino acids, is an essential component of the hypothalamus-pituitary-adrenal (HPA) axis. It stimulates the secretion of pituitary adrenocorticotropin hormone (ACTH) and in turn the release of corticosterone or cortisol from the adrenal glands [1]. The cellular effects of CRF family peptides are mediated through CRF receptor subtypes 1 (CRFR1) and 2 (CRFR2) [2]. Over the past few decades, many studies have proved that CRF can regulate feeding behavior. The administration of CRF into the central nervous system suppresses feed intake and increases energy mobilization in mammals and birds [3,4].

Ghrelin was extracted from rat stomachs approximately 20 years ago as the endogenous ligand to the growth hormone secretagogue receptor (now termed GHSR1a) and thus an efficient stimulator of pituitary GH release in rats and humans [5]. Ghrelin plays an instrumental role in the regulation of feed intake and energy balance in various vertebrate groups. In mammals, ghrelin generally promotes feed intake, body weight gain, and obesity through the central and peripheral modes of action [6]. In avian species, the effects of ghrelin treatment on feed intake depend on the dose, species, and route of administration. Central ghrelin injections suppress feed intake [7]. This effect may be mediated through the central anorexigenic CRF system, as shown in neonatal chicks [7].

However, whether CRF can reduce poultry feed intake via ghrelin has not been fully explored. Therefore, the present study was designed to explore the effects of intracerebroventricular (ICV) injection of CRF on the ghrelin and CRF receptors in the hypothalamus and intestines of broiler chickens.

## MATERIALS AND METHODS

### Animals and experimental design

The protocol for the current experiment was received and approved by the Institutional Animal Care and Use Committee at Shandong Agriculture University (IACUC:0026-1896).

A total of 64 male broiler chickens (Arbor Acres, *Gallus gallus domesticus*) with similar weights (Shandong Dabao Poultry Co., Ltd., Tai’an, Shandong, China) were used. The cage breeding method was performed, and the feeding temperature was 35°C. The broiler chickens had access to natural ventilation, and the relative humidity was maintained at 55% to 65%. Raising feed refers to basic feed prepared according to the nutrient requirement of NRC (1994) broiler chickens. The composition and nutritional level are shown in Table 1. The broiler chickens had free access to feed intake and water. Two trials were conducted.

*Trial 1*: Male broiler chickens received normal feeding, and 32 of them had similar weights (150±5 g) on the 7th day. After each broiler chicken received a third-ventricular cannula as previously described [8,9], the 32 selected broiler chickens were randomly divided into four groups and fed in single cages. Three days following surgery, at 8 a.m. on the 10th day, ICV injection of CRF or normal salt solution was carried out. The broiler chickens were divided into the 5, 10, and 20 μg and control group (normal salt solution) according to the CRF injection dose. After injection, the feed intake was calculated at the 30th, 60th, 90th, and 120th minutes.

*Trial 2*: The trial was carried out at a dose of 10 μg for 120 min, in which the most obvious inhibition on feed intake occurred. Therefore, in trial 2, broiler chickens were divided into the 10 μg CRF and control group (physiological saline). After injection at the 120th minute, the expression of the associated mRNA in the hypothalamus and intestines was measured.

### Sample collection and parameters determination

The feed intake of each broiler was recorded within 2 h after injection. Tissue samples were obtained from the hypothalamus, duodenum, jejunum, and ileum. The tissue samples were washed with ice-cold sterilized saline, cooled down in liquid nitrogen, and stored at −80°C for further analysis. Total RNA was extracted from the hypothalamus, duodenum, jejunum, and ileum according to the instructions of the Animal Tissue/Cell RNA extraction kit (Beijing Kangwei Century Biotechnology Co., Ltd., Beijing, China), and the total RNA concentration and purity were detected at the wavelength of 260 nm by using a micro-ultraviolet spectrophotometer (DS-11; DeNovix, Wilmington, DE, USA).

RT reactions (10 μL) consisted of 500 ng total RNA, 5 mmol/L MgCl_2_, 1 μL of RT buffer, 1 mmol/L dNTP, 2.5 U AMV, 0.7 nmol/L oligo (dT), and 10 U ribonuclease inhibitor. The reverse transcription reaction parameters were as follows: 25°C for 10 min, 55°C for 30 min, and 85°C for 5 min. Real-time polymerase chain reaction (PCR) analysis was conducted using the Applied Biosystems 7500 real-time PCR system (Applied Biosystems, Foster, CA, USA). Each RT-reaction served as a template in a 20 μL PCR reaction containing 0.2 μmol/L of each primer and SYBR green master mix (Takara, Otsu, Japan). Primer-set sequences are described in Table 2. Real-time PCR reactions were performed at 95°C for 10 s, followed by 40 cycles at 95°C for 5 s and 60°C for 40 s. SYBR green fluorescence was detected after each cycle to monitor the amount of PCR product. For the calculation of the efficiency of qPCR primers, a standard curve was made in five-fold dilutions, and its slope was used to calculate efficiency.

The relative amount of mRNA for a gene was calculated as previously described [10]. The mRNA levels of these genes were normalized to glyceraldehyde 3-phosphate dehydrogenase (GAPDH) levels (ΔCT). The ΔCT was calibrated against an average of control broiler chickens. The linear amount of target molecules relative to the calibrator was calculated using the 2^−ΔΔCT^ method. All gene transcription results are reported as the *n*-fold difference relative to the calibrator. The specificity of the amplification product was verified by electrophoresis on a 0.8% agarose gel and by DNA sequencing.

### Statistical analysis

All data were subjected to one-way analysis of variance to determine the main effect of the treatment (SAS institute, 1998). When the main effect of treatment was significant, differences between means were assessed by Duncan’s multiple range analysis.

## RESULTS

### Effect of ICV injection with CRF on the feed intake of broiler chickens

**Figure 1:**
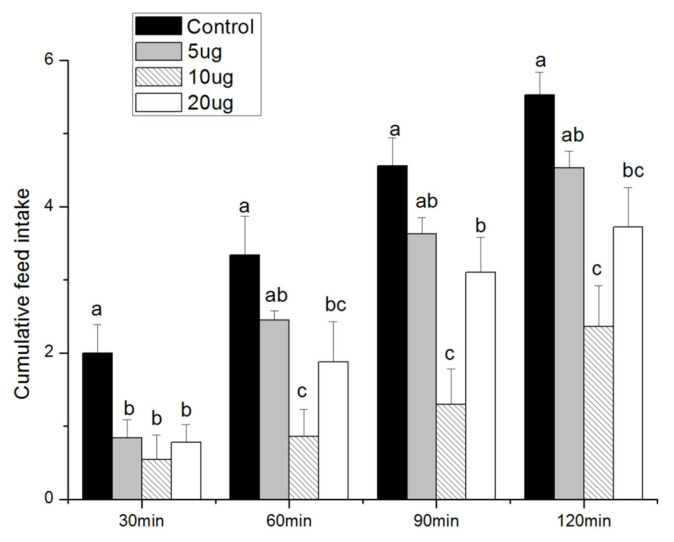
Effect of intracerebroventricular injection of corticotropin-releasing factor (CRF) on accumulative broiler feed intake within 2 h post-injection. Control = control group (basal diet); 5 μg = 5 μg/kg body weight; 10 μg = 10 μg/kg body weight; 20 μg = 20 μg/kg body weight; ^a–c^ Values with different letters differ significantly (p<0.05); n = 8.

The cumulative amount of feed intake was significantly lower than that of the control group (p<0.05) at CRF dose of 10 or 20 μg and at 30, 60, 90, and 120 min after injection (Figure 1). The lowest feed intake was obtained at a 10 μg CRF dose.

### Effects of ventricular injection with CRF on the hypothalamus and intestinal-related genes in broiler chickens

Treatment with 10 μg CRF increased ghrelin and CRF1 expression (p<0.05) but had no significant effect on the mRNA levels of GHSR-1a and CRFR2 (p>0.05) in the hypothalamus.

**Figure 3:**
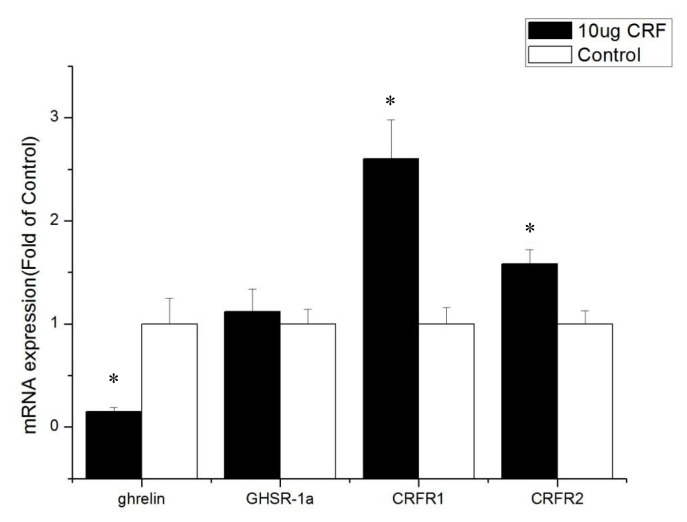
Effect of intracerebroventricular injection of corticotropin-releasing factor (CRF) on genes in the duodenum of broiler chickens. Control = control group (basal diet); 10 μg = 10 μg/kg body weight; n = 8. * p<0.05 (compared with control).

The ICV injection with 10 μg CRF significantly reduced the abundance of ghrelin mRNA expression (p<0.05), increased CRFR1 and CRFR2 mRNA levels (p<0.05), and had no significant effect on the mRNA level of GHSR-1a (p>0.05) in the duodenum (Figure 3).

**Figure 4:**
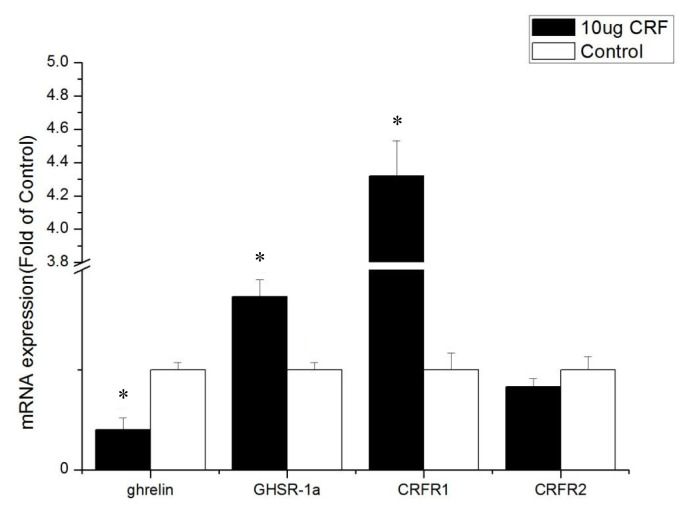
Effect of intracerebroventricular injection of corticotropin-releasing factor (CRF) on genes in the jejunum of broiler chickens. Control = control group (basal diet); 10 μg = 10 μg/kg body weight. n = 8. * p<0.05 (compared with control).

The ICV injection with 10 μg CRF significantly reduced the abundance of ghrelin mRNA levels (p<0.05) in the jejunum. The CRF (10 μg) injection increased the GHSR-1a and CRFR1 mRNA expression (p<0.05, Figure 4).

**Figure 5:**
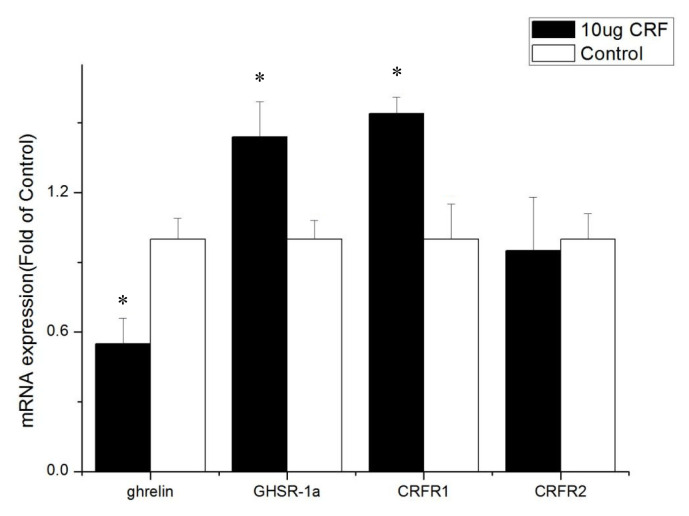
Effect of intracerebroventricular injection of corticotropin-releasing factor (CRF) on genes in the ileum of broiler chickens. Control = control group (basal diet); 10 μg = 10 μg/kg body weight. n = 8. * p<0.05 (compared with control).

Ghrelin mRNA levels in the ileum from CRF-injected chickens decreased compared with the control (p<0.05, Figure 5). CRF injection increased the mRNA levels of GHSR-1a and CRFR1 (p<0.05). However, the CRFR2 mRNA levels in the ileum and jejunum were not altered by CRF injection (p>0.05), showing their less important role in feed intake.

## DISCUSSION

### Effect of ICV injection with CRF on the feed intake of broiler chickens

The CRF, a 41-amino acid peptide secreted by parvocellular neurons of the paraventricular nuclei, is a physiological regulator of ACTH release invertebrates. The CRF has been implicated in several physiological functions in mammals, including stress responses [11], immune function [12], and feeding behavior [13]. The CRF administration into the central nervous system reduces feed intake and mediates the stress-induced suppression of feed intake [14]. The CRF suppresses feed intake in rats after ICV [15]. The CRF decreases feed intake after ICV injection in chickens [16]. The present study found that intraventricularly administered CRF was effective in decreasing feed intake at doses of 10 and 20 μg at 30, 60, 90, and 120 min. These results are consistent with previous data from rat or chicken. The result also indicates that the anorexigenic degree of CRF was influenced by injection dose. The lowest value of feed intake was observed after injection with 10 μg CRF. Therefore, the best effect of suppressing feed intake could be obtained by injection with 10 μg CRF.

The feed intake was increased with 20 μg compared with 10 μg, but no significant difference was observed between the two treatments, except at 90 min. In general, the effects of 10 and 20 μg CRF on feed intake are similar. At 20 μg of CRF, the slightly increased feed intake caused damage to the body. Therefore, the anorexigenic effect of CRF at 20 μg was not more remarkable than that at 10 μg.

The ICV injection of CRF inhibition gastric emptying [17] and hyperglycemia [18], which reduce feed intake. Specifically, the slowing of gastric emptying causes the accrual of feed in the stomach and the transmission of satiety signals to the brain [19]. Therefore, in the present study, the decrease in feed intake in broiler chickens after ICV injection with CRF is related to the decreased rate of gastric emptying and the transmission of satiety signals to the brain.

### Effects of ICV injection with CRF on the ghrelin system and CRFR1 and CRFR2

The ICV injection with CRF (10 μg) significantly increased the mRNA level of ghrelin in the hypothalamus of the broiler chickens, but the expression of ghrelin mRNA in the intestines (duodenum, jejunum, and ileum) decreased significantly. Considering that the hypothalamus integrates a range of different peripheral and central signals, it is the ultimate regulator of feed intake, that is, the hypothalamus is the main area of appetite regulation [20]. Ghrelin is produced in an animal’s stomach, released into the bloodstream [21], and passively transported into the central nervous system [22]. The high expression of ghrelin in the hypothalamus can affect the feed intake of broiler chickens. Ghrelin inhibits the feed intake of broiler chickens [23,24], and Ghrelin’s anorexia effect on newborn broiler chickens is mediated by the CRF system [7]. Our experiments show that ICV injection with CRF increased the expression of ghrelin mRNA in the hypothalamus. Then, ghrelin in the hypothalamus inhibited the feed intake of broiler chickens. Moreover, the reduction of ghrelin mRNA level in the intestine may have resulted from the negative feedback of ghrelin itself. These results suggest that the appetite-suppressing effect of CRF occurs in conjunction with ghrelin.

GHSR-1a, also known as the ghrelin functional receptor, is a G protein-coupled receptor expressed in many central and peripheral tissues but predominantly in the hypothalamus-pituitary unit [25], and this distribution perfectly matches the first reports of the ghrelin-induced regulation of energy balance and GH release [6,26]; GHSR-1a acts as the functional receptor of ghrelin, indicating that ghrelin may stimulate the expression of β-casein via GHSR-1a [27]. After ghrelin is injected peripherally at a large dose, it can pass through the blood–brain barrier and bind with GHSR in the hypothalamus, resulting in loss of appetite [28]. In this experiment, ICV injection with CRF (10 μg) significantly increased the gene expression of GHSR-1a in the intestines and may have been caused by the activation of the HPA axis. The CRF can stimulate the secretion of ACTH and in turn the release of corticosterone or cortisol from the adrenal glands [1]. Kageyama et al [29] found that GHSR1 mRNA levels are stimulated both directly by glucocorticoids and indirectly by ghrelin. Accordingly, increased GHSR1a mRNA levels in the intestines were caused by glucocorticoid, which was stimulated by CRF. The GHSR protein binds to ghrelin to play a critical role in the central and peripheral regulation of growth hormone secretagogues, feed intake, and energy homeostasis [30]. In the present study, real-time PCR assays show that the GHSR-1a expression levels in the jejunum and ileum of CRF-injected broiler chickens were high, indicating that GHSR-1a might positively regulate the feed intake for broiler chickens administrated with CRF has decreased feed intake.

Kitazawa et al [31] reported that ghrelin can stimulate the contraction of the upper (esophagus and crop) and lower (colon) parts of the gastrointestinal (GI) tract and has only a weak stimulatory effect on the middle part (proventriculus, duodenum, and jejunum) of the GI tract. Therefore, in birds, the effect of ghrelin on different parts of GI is not completely the same. The expression of GHSR-1a (known as the ghrelin functional receptor) in the duodenum was not increased by ghrelin, and the condition was enhanced by CRF injection, whereas the expression of GHSR-1a in the jejunum and ileum increased.

The cellular effects of CRF family peptides are mediated by CRFR1 and CRFR2 [32]. CRFR1 and CRFR2 are widely distributed in the central nervous system, and each receptor plays a unique role in CRF-related systems. CRF mainly binds to CRFR1, and CRFR1 shows high affinity for CRF, while CRFR2 shows low affinity [33]. CRFR1 mediates the effects of CRF, including pituitary ACTH release in rats [34]. Hotta et al [35] showed that brain CRF is involved in the inhibition of feeding behavior and modulation of locomotor activity in emotional stress through CRFR1. Upon activation, CRFR1 can inhibit the feed intake of young *Xenopus laevis* [36]. However, Stengel et al [32] showed that the CRF-CRFR2 system is the primary signaling pathway that mediates the anorexigenic effect of CRF. Some experiments show that CRF mainly activates CRFR1 and CRFR2 but to a less extent [37]. This result is consistent with the results of the current experiment. The present study found that CRFR1 expression increased in the hypothalamus and intestines after injection with CRF, whereas CRFR2 expression was only improved in the duodenum, indicating that CRF inhibits the feed intake behavior of broiler chickens mainly through interaction with CRFR1.

## CONCLUSION

Treatment with 10 μg CRF increased ghrelin expression in the hypothalamus and CRFR1 expression in the small intestine, which was related to suppressed feed intake of broiler chickens.

## Figures and Tables

**Figure 2:**
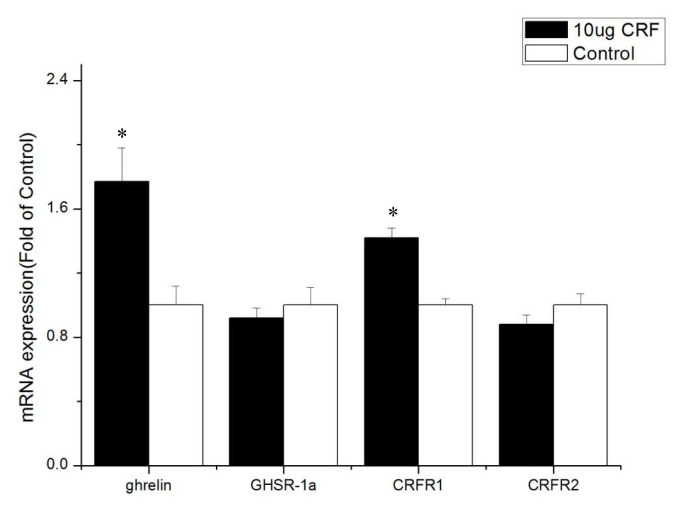
Effect of intracerebroventricular injection of corticotropin-releasing factor (CRF) on genes in hypothalamus of broiler chickens. Control = control group (basal diet); 10 μg = 10 μg/kg body weight. n = 8 * p<0.05 (compared with control).

**Table 1:** Composition and nutrient levels of the basal diet (air-dry basis %)

Items	Content (%)
Ingredients	
Corn	52.19
Soybean oil	5.13
Soybean meal	38.93
Limestone	1.46
CaHPO_4_	0.80
NaCl	0.30
L-Lysine·H_2_SO_4_	0.27
DL-Methionine	0.30
L-Threonine	0.10
Phytase (5,000 IU/g)	0.02
Muti-vitamins^1)^	0.20
Muti-mineral^1)^	0.20
Choline chloride	0.10
Total	100.00
Nutrient levels^2)^	
Crude protein	21.50
ME (MJ/kg)	12.81
Ca	0.95
Non-phytate phosphorus	0.44
Lysine	1.19
Methionine	0.59
Met+Cys	0.87
Threonine	0.76
Tryptophane	0.22

1) Multi-vitamins and -minerals provided the following per kg of the diet: 9,000 IU Vitamin A, 2,000 IU Vitamin D_3_, 11.0 IU Vitamin E, 1.00 mg Vitamin K, 1.2 mg thiamine, 5.80 mg riboflavin, 66.0 mg niacin, 10.0 mg pantothenic, 2.6 mg pytidoxine, 0.2 mg biotin, 0.70 mg folic acid, 0.012 mg Vitamin B_12_, 100 mg Mn, 75.0 mg Zn, 80.0 mg Fe, 0.65 mg I, 8.00 mg Cu, and 0.35 mg Se.

2) Nutrient levels were calculated values.

**Table 2:** Gene-specific primers of related genes

Gene	Gene bank	Primers sequences (5′ → 3′)	Product size (bp)
*GAPDH*	NM_204305	F: 5′-ACATGGCATCCAAGGAGTGAG-3′	266
		R: 5′-GGGGAGACAGAAGGGAACAGA -3′	
*CRF*	NM_001123031	F: 5′-CTCCCTGGACCTGACTTTCC-3′	86
		R: 5′-TGTTGCTGTGGGCTTGCT-3′	
*Ghrelin*	AB075215	F: 5′-CCTTGGGACAGAAACTGCTC-3′	203
		R: 5′-CACCAATTTCAAAAGGAACG-3′	
*GHSR-1a*	AB095995.1	F: 5′-TTTTTCCTGCCCGTATTCTG-3′	290
		R: 5′GCTTGGTGCTGGAGAGTCTT-3′	
*CRFR1*	NM_204321.1	F: 5′-CCTCACCTATTCCACCGACAAG-3′	134
		R: 5′-GCTTCCCAAACCAGCACTTCT-3′	
*CRFR2*	NM_204454.1	F: 5′-TGCTCCAAATGATAGACCACAA-3′	117
		R: 5′-AGCCTTCCACAAACATCCAGAA-3′	

*GAPDH*, glyceraldehyde phosphate hydrogenase; *CRF*, corticotropin-releasing factor; *GHSR-1a*, growth hormone secretagogue receptor-1a; *CRFR1*, corticotropin-releasing factor receptor-1; *CRFR2*, corticotropin-releasing factor receptor-2.
